# Assessing the cytotoxicity of aerosolized carbon black and benzo[a]pyrene with controlled physical and chemical properties on human lung epithelial cells

**DOI:** 10.1038/s41598-023-35586-7

**Published:** 2023-06-08

**Authors:** Youngri Ryu, Soonjong Roh, Young Soo Joung

**Affiliations:** Department of Mechanical Systems Engineering, Sookmyung Women’s University, 100, Cheongpa-ro 47-gil, Yongsan-gu, Seoul, Republic of Korea

**Keywords:** Environmental sciences, Risk factors, Mechanical engineering, Mechanisms of disease

## Abstract

Atmospheric particulate matter (PM) is a complex mixture of hazardous particles containing hundreds of inorganic and organic species. Organic components, such as carbon black (CB) and benzo[a]pyrene (BaP), are known to exhibit diverse genotoxic and carcinogenic effects. The toxicity of CB and polycyclic aromatic hydrocarbons has been well studied, however the combined toxicity is much less understood. A spray-drying system was used to control the size and chemical composition of PMs. PMs were prepared by loading BaP on three different sized CBs (0.1 μm, 2.5 μm, and 10 μm) to obtain BaP-unloaded CB (CB_0.1_, CB_2.5_, and CB_10_) and BaP-loaded CB (CB_0.1_–BaP, CB_2.5_–BaP, and CB_10_–BaP). We analyzed cell viability, levels of oxidative stress, and pro-inflammatory cytokines using human lung cells (A549 epithelial cells). Cell viability decreased when exposed to all PMs (PM_0.1_, PM_2.5_, and PM_10_), regardless of the presence of BaP. The increase in PM size due to BaP-adsorption to CB resulted in insufficient toxic effects on human lung cells compared to CB alone. Smaller CBs reduced cell viability, leading to reactive oxygen species formation, which can cause damage to cellular structures deliver more harmful substances. Additionally, small CBs were predominant in inducing the expression of pro-inflammatory cytokines in A549 epithelial cells. These results indicate that the size of CB is a key factor that immediately affects the inflammation of lung cells, compared to the presence of BaP.

## Introduction

Airborne particulate matter (PM) is a heterogeneous mixture of solid and liquid particles suspended in the atmosphere and is associated with air pollution and adverse human health^1^. In recent years, approximately 91% of the world's population has lived in areas where air quality is below the World Health Organization (WHO) recommendations due to air pollution. In particular, in East Asia, exposure to PM_2.5_ caused 1.14 million premature deaths from respiratory and cardiovascular diseases, accounting for 27% of the annual early mortality^2^. Recent studies have consistently shown correlations between elevated outdoor PM levels and wide-ranging deleterious effects on human health, including early mortality, and pulmonary and cardiovascular diseases^3^. Furthermore, particles originating from the outdoors occupy a significant amount of indoor particle composition, adversely affecting indoor air quality^4^. Thus, continuous research on outdoor particles is essential to prevent diverse diseases caused by exposure to outdoor particles and to improve the indoor environment.

PM can be classified into three aerodynamic diameter levels according to the deposition and penetration ability of particles in the human respiratory tract: $\le$ 10 μm, $\le$ 2.5 μm, and $\le$ 0.1 μm (PM_10,_ PM_2.5,_ and PM_0.1_)^5^. Inhalable particles, including ultrafine particles (UFPs) that have a diameter of less than 0.1 µm, are a major component of air pollution in most cities. Inhalable particles, especially ultrafine particles (UFPs), are major pollutants in most cities^4^. Most particles larger than 10 μm in diameter are filtered out of the upper respiratory tract via mucociliary clearance^6^; therefore, particles larger than PM10 are prone to remain in the upper respiratory tract because they deposit rapidly in the nasopharyngeal membranes^5^. Because particles with a diameter of 2.5 μm or less have a relatively larger surface area than PM_10_, they can transport various toxic substances to all organs through the respiratory tract. The factors that affect the transport of UFPs to the bronchi end include particle size, ambient concentration, respiratory behavior (breathing rate, tidal volume, and depth of breathing), and physiological factors. Of these factors, particle size is the most important, as UFPs with diameters smaller than 0.1  μm cannot be effectively filtered by systems such as nose hairs^7,8^. UFPs smaller than 0.1 μm can reach the end of the bronchi through filtration of nose hairs, penetrate the alveoli, and eventually enter the blood circulation system^9^. Therefore, particle size is directly related to the potential to cause health problems^10^.

PMs vary in characteristics depending on the emission source, origin, and weather conditions of the geographic location^11^. In addition, most hazardous particles are generated by anthropogenic activities such as incineration, vehicle exhaust, domestic heating, and incomplete combustion of organic fuels^12^. Artificially generated noxious substances are composed of organic carbon and inorganic elements. Carbon blacks (CB) and polycyclic aromatic hydrocarbons (PAHs) are the main constituents that induce toxicological effects and inflammatory responses^13^. CB is an elemental particle produced by industrial partial combustion that adsorbs harmful chemicals, transports them into the circulatory system, and for particle sizes below 2.5 μm, it is known to penetrate cellular organelles^14–16^ even enters cellular organelles. Benzo[a]pyrene (BaP), a PAH component, arises from incomplete combustion of organic matter^13,17^. Moreover, several studies have reported that exposure to BaP increases DNA modification, resulting in diverse genotoxic and carcinogenic effects^18,19^. To investigate the physical and chemical effects of PMs on human lung cells, various PAH components were coated on single-sized carbon nanoparticles and compared^20,21^; however, consistent experimental results were not obtained depending on the target cell and test system. Additionally, the biological reactions that occur when different physicochemical properties of particles are combined is still up for debate^20^. Therefore, it is necessary to investigate the biological effects of PMs consisting of CB and BaP on human health, depending on their physicochemical properties^21–23^. Most studies on the toxicity of PMs have not considered the independent control of the physical and chemical properties of the particles. In addition, these studies collected ambient particles and used them without further treatment^7,24^.

Previous epidemiological and toxicological studies suggest that health effects associated with exposure to PMs are related to various physicochemical properties^14^. This study examined the effect of the physicochemical properties of PM consisting of CB and BaP on inflammation in human lung epithelial cells. To understand the inflammatory response and cytotoxicity mechanism of PM in lung cells, it is necessary to analyze the physicochemical characteristics of PMs. The toxicity of PMs composed of CB and PAHs has been well studied individually, but the combined toxicity is much less understood. To overcome these limitations, more research is needed to understand the combined toxicity CB and BaP in PMs and develop effective strategies to mitigate their adverse health effects^25^. Additionally, the physical and chemical properties of PMs must be controlled independently^26^. Here, PMs are prepared using an aerosolization technique to control the particle size and chemical composition by loading BaP on CBs of three different sizes (PM_10,_ PM_2.5,_ and PM_0.1_). We elucidated the complex effects of the physicochemical properties of PMs composed of CBs, with and without BaP, on the inflammatory response. We showed that the size of CBs is a decisive factor in the inflammatory response in lung cells. In addition, for the first time, we demonstrated a new role of BaP in changing the physical properties of PMs during the aerosolization process, resulting in different inflammatory responses depending on the size of CB. We believe that our findings will provide a more detailed understanding of the toxicological response to the exposure of PMs consisting of CB and BaP in terms of their physical and chemical properties.

## Methods

### Materials

CB (0.1 μm and 2.5 μm; CB_0.1_ and CB_2.5_), BaP, sodium bicarbonate, dimethyl sulfoxide, and trypan blue solution were purchased from Sigma-Aldrich (Saint-Louis, USA). CB_10_ (10 μm CB) was purchased from New Material Industry Co., Ltd (Seoul, South Korea). Anhydrous ethyl alcohol (99.5%) was purchased from Daejung Chemicals & Metals Co., Ltd (Siheung, South Korea). RPMI-1640 supplemented with L-glutamine (12-702F) was purchased from Lonza (Basel, Switzerland). Dulbecco's phosphate-buffered saline (LB001-02), fetal bovine serum (12483-020), and HEPES buffer solution (15630-080) were purchased from Welgene (Gyeongsan, South Korea).

### Preparation of PM

**Figure 1:**
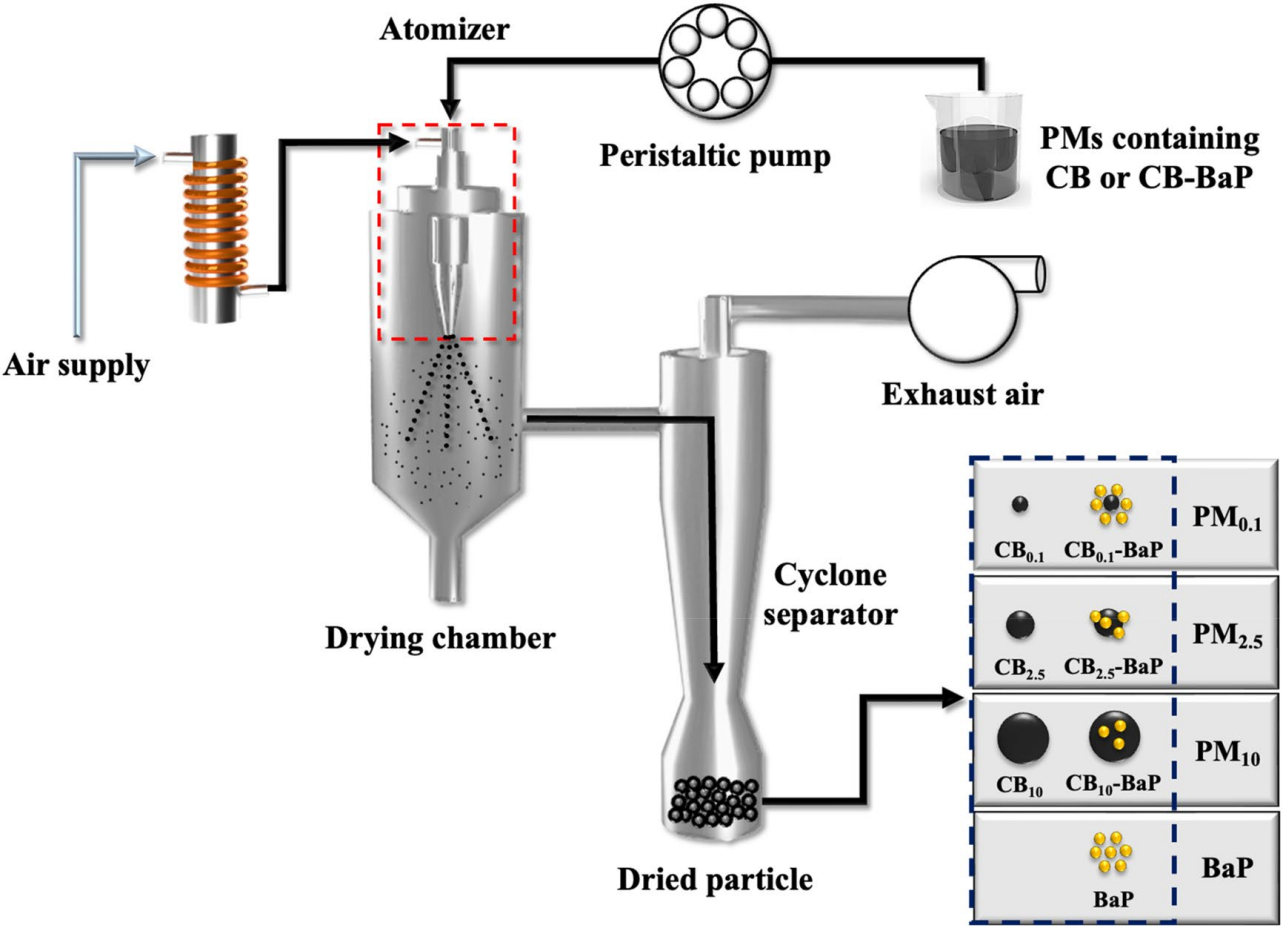
Schematic diagram of the fabrication method of particulate matters (PMs). Seven different types of PMs were made through a spray drying procedure based on the aerosolization of droplets containing carbon black (CB) with/without Benzo[a]pyrene (BaP). The black dotted box indicates the seven types of PMs produced with three different sizes of CB (CB_0.1_, CB_2.5_, and CB_10_), CB with BaP (CB_0.1_–BaP, CB_2.5_–BaP, and CB_10_–BaP), and BaP only (BaP) at 121 °C.

As shown in Fig. 1, a spray-drying system was used to produce PMs with different sizes and chemical compositions. Three different sized CBs (0.1μm, 2.5 μm, and 10μm) and BaP were used to prepare BaP-unloaded CB (CB_0.1_, CB_2.5_, and CB_10_) and BaP-loaded CB (CB_0.1_–BaP, CB_2.5_–BaP, and CB_10_–BaP). Before the spray-drying process, CB and BaP were dissolved in 20% ethanol at a ratio of 100:2.5. The CB–BaP group was prepared by diluting 2 g of CB and 50 mg of BaP in ethanol, followed by the addition of 160 ml of D.W. The BaP group was prepared by diluting 2 g of BaP in ethanol and then adding 160 ml of distilled water (D.W). The mixture was evenly dispersed using a magnetic stirrer for 10 min. The well-dispersed solution was supplied to a spray dryer (OLT-SD8000ST, Xiamen Ollital Technology Co., Ltd.) by using a peristaltic pump. In the drying chamber, high-temperature (121 °C) drying air passing through an electric heater flowed co-currently with the spray droplets to eliminate moisture. Dried PMs were collected in a tank from the bottom of the cyclone. As the final standard stock solution, The PMs were dispersed in 100 μl of dimethyl sulfoxide (DMSO) and diluted with 900 μl of cell medium to obtain a final volume of 1 ml and a DMSO concentration of 0.1%. PMs were dispersed in 100 μl of DMSO and diluted with 900 μl of cell medium to obtain a final DMSO concentration of 0.1%. Subsequently, the final standard solution was stored at -20 °C to avoid deformation of the PM characteristics.

### Physicochemical analysis

#### Particle size distribution and concentration

The particle size and morphology of the PMs according to the concentration were evaluated using microscopy (Eclipse Ni-U, Nikon). Size distribution measurements of the prepared PMs were performed immediately after the suspension reached at room temperature (24 °C) using a particle size analyzer (90 Plus, Brookhaven). The particle size was quantified using dynamic light scattering (DLS), and the particle size range of DLS was set from 0.3 nm to 66 μm. The cuvettes were filled with 3 ml of the particle suspension and measured three times to obtain the average particle size with the standard derivative.

#### CB and BaP analysis

BaP, one of the PAH components, was spray-dried CB-BaP and analyzed by gas chromatography–mass spectrometry (GC–MS) equipped with DB-5MS UI columns (Model 8890A 5977 B GC–MS, Agilent, USA) to quantify the BaP components. After the pretreatment process, all samples were analyzed through a 0.45 μm pore filter.

#### Scanning electron microscopy (SEM) analysis

SEM (JSM-7600F, JEOL) was performed to visualize the surface morphology of the CB and CB-BaP particles by size. The PMs were evenly deposited on the carbon tape without agglomeration. The excess particles were removed by blowing the surface. The samples were coated via platinum sputtering and observed at an accelerating voltage of 8–10 kV.

### Biological analysis

#### Human lung cell culture (A549 cells)

A549 cells, an adenocarcinomic human alveolar basal epithelial cell line, were purchased from the Korean Cell Line Bank (KCLB). The cells were cultured in KCLB medium (RPMI 1640 supplemented with L-glutamine, 25 mM HEPES, 25 mM NaHCO_3_ and heat-inactivated 10% fetal bovine serum) in a humidified incubator (MCO-170AIC-PK, PHCbi) at 37 °C in a 5% CO_2_ atmosphere. The medium was changed every 3–4 days to ensure sustainable cell growth. When the cell growth density in the cell culture flask reached 5.0 × 10^6^ cells/ml, they were harvested with TrypLE™ Express (12604-021, Gibco) and collected by centrifugation at 1200 rpm for 5 min. The collected cells were diluted with KCLB medium and seeded in 96-well culture plates (1 × 10^6^ cells/ml per well) to expose them to the different sized PMs.

#### PMs exposure protocol

To expose human lung cells to PMs for toxicity assessment, experimental groups were set according to the size and concentration of PMs with or without BaP, as shown in Table 1. Before the cells were exposed to PMs, the final concentration of the frozen particle solution was maintained at room temperature. The particle solution was sufficiently vortexed and diluted in culture medium to obtain final exposure concentrations of 10 μg/ml (low), 100 μg/ml (mid), and 500 μg/ml (high). Prior to beginning the experiments outlined in the present study, after exposing A549 cells to PM_2.5_ in the range of 0.1–500 μg/ml, it was confirmed that the most oxidative stress and cell apoptosis occurred at a concentration of 500 μg/ml, and three optimal concentrations were set through preliminary experiments^27^.

**Table 1:** Experimental details for the biological analysis.

Physical classification	Chemical classification	PM composition	PM concentration
Control	Control	DMSO + media	
PM_0.1_	(CB_0.1_)_low_	(DMSO + media) + carbon black (0.1 µm)	10 µg/ml
	(CB_0.1_)_mid_	(DMSO + media) + carbon black (0.1 µm)	100 µg/ml
	(CB_0.1_)_high_	(DMSO + media) + carbon black (0.1 µm)	500 µg/ml
	(CB_0.1_ + BaP)_low_	(DMSO + media) + carbon black (0.1 µm) + Benzo[a]pyrene	100 µg/ml
	(CB_0.1_ + BaP)_mid_	(DMSO + media) + carbon black (0.1 µm) + Benzo[a]pyrene	100 µg/ml
	(CB_0.1_ + BaP)_high_	(DMSO + media) + carbon black (0.1 µm) + Benzo[a]pyrene	500 µg/ml
PM_2.5_	(CB_2.5_)_low_	(DMSO + media) + carbon black (2.5 µm)	10 µg/ml
	(CB_2.5_)_mid_	(DMSO + media) + carbon black (2.5 µm)	100 µg/ml
	(CB_2.5_)_high_	(DMSO + media) + carbon black (2.5 µm)	500 µg/ml
	(CB_2.5_ + BaP)_low_	(DMSO + media) + carbon black (2.5 µm) + Benzo[a]pyrene	10 µg/ml
	(CB_2.5_ + BaP)_mid_	(DMSO + media) + carbon black (2.5 µm) + Benzo[a]pyrene	100 µg/ml
	(CB_2.5_ + BaP)_high_	(DMSO + media) + carbon black (2.5 µm) + Benzo[a]pyrene	500 µg/ml
PM_10_	(CB_10_)_low_	(DMSO + media) + carbon black (10 µm)	10 µg/ml
	(CB_10_)_mid_	(DMSO + media) + carbon black (10 µm)	100 µg/ml
	(CB_10_)_high_	(DMSO + media) + carbon black (10 µm)	500 µg/ml
	(CB_10_ + BaP)_low_	(DMSO + media) + carbon black (10 µm) + Benzo[a]pyrene	10 µg/ml
	(CB_10_ + BaP)_mid_	(DMSO + media) + carbon black (10 µm) + Benzo[a]pyrene	100 µg/ml
	(CB_10_ + BaP)_high_	(DMSO + media) + carbon black (10 µm) + Benzo[a]pyrene	500 µg/ml
BaP	(BaP)_low_	(DMSO + media) + Benzo[a]pyrene	10 µg/ml
	(BaP)_mid_	(DMSO + media) + Benzo[a]pyrene	100 µg/ml
	(BaP)_high_	(DMSO + media) + Benzo[a]pyrene	500 µg/ml

The experimental groups were determined by particle size, concentration, and presence of benzo[a]pyrene (BaP). PM is a group classified by physical properties and CB + BaP is a group classified by chemical properties. The particle concentration is represented as low, mid, and high. All particles were dispersed in DMSO, and the final concentration was adjusted to 0.1% DMSO with the A549 cell culture medium. The cytotoxicity bioassays were analyzed for all concentrations (low, mid, and high) of the prepared particles. The other bioassays (ROS and ELISA) were performed at high concentrations.

#### MTT assay for cell viability

The cytotoxicity of PMs was assessed using the 3-[4,5-dimetho-thiazol-2-yo]-2,5-diphenyl tetrazolium bromide (MTT) assay, which uses a cell proliferation kit (11465007001, Roche) based on the principle of measuring surviving cells through formazan color. A549 cells were seeded in a 96 well at a density of 5.0 × 10^4^ cells/well in triplicate. After 24 h of exposure to PMs, the cells were incubated with 10 μl of MTT labeling reagent (11465007001, Roche) for 24 h. After formazan salt was formed by viable cells in a chemical reaction, 100 μl of solubilization solution (11465007001, Roche) was added to each well to dissolve the formazan. After overnight incubation, absorbance was measured using a microplate reader (SpectraMax i3x, Molecular Devices) at 570 nm. The cell viability was calculated using the following Eq. (1). *A*_*s*_ means the OD value of the PM-treated group, *A*_*b*_ refers the OD value of blank group, and *A*_*c*_ exhibits the MOD value of control group.

1 $${\text{Cell}}\;{\text{viability}}\left( \% \right) = \frac{{A_{s} - A_{b} }}{{A_{c} - A_{b} }} \times 100\%$$

#### Detection of reactive oxygen species (ROS)

Intracellular ROS levels were measured using the 2,7-dichlorodihydro-fluorescein diacetate (DCFDA) assay, which was applied to a cellular ROS assay kit (ab113851, Abcam) based on the principle of analyzing the byproducts of cellular oxidative metabolism. A549 cells were in 96 well plates at a density of 2.5 × 10^4^ cells/well in triplicate. The cell culture medium was discarded, and the cells were washed with 100 μl pre-warmed 1 × buffer (ab113851, Abcam). The cells were then incubated with 20 μM DCFDA solution (ab113851, Abcam) at 37 °C for 45 min in the dark. After removing the buffer, 100 μl of the prepared PMs were exposed for 1 h, 3 h, and 6 h. Fluorescence was measured using a microplate reader (SpectraMax i3x, Molecular Devices) at Ex/Em = 485/535 nm.

#### ELISA assay

The levels of proinflammatory cytokines in the supernatant were assessed using Quantikine® ELISA kits (S6050, R&D Systems). The levels of two cytokines (Interleukin-6; IL-6 and IL-8) were quantified according to the procedure recommended by the ELISA kit. A549 cells were seeded in triplicate in a 96-well plate at a density of 5.0 × 10^4^ cells/well. After 24 h of exposure to PMs, cell culture supernatants were removed by centrifugation. Cell supernatants were transferred to a 96-well microplate coated with antibodies specific for human IL-6 and IL-8. After removing any non-specific bound antibodies by washing, polyclonal antibodies conjugated to an enzyme (horseradish peroxidase) were added to each well. A color reagent containing hydrogen peroxide and chromogen was then added. Finally, after sufficient binding to the substrate, the color changed to indicate the amount of sample antigen. IL-6 and IL-8 levels were quantified using a microplate reader (SpectraMax i3x, Molecular Devices) at 450 nm.

### Statistical analysis

All data are expressed as mean values ± standard error of the mean. The minimum number of replicates for all measurements was at least three times. SPSS (SPSS version.25, Chicago, IL, USA) was used for statistical analysis. Differences between experimental groups were compared using one-way ANOVA combined with an independent sample t-test. In all cases, when the p value was less than 0.001 (*p* < 0.001), the difference was considered significant.

## Results

### Physicochemical analysis of PMs

#### Physical characteristics

Physicochemical analysis was conducted to confirm the size, shape, and concentration of PMs prepared using the spray drying method.

**Figure 2:**
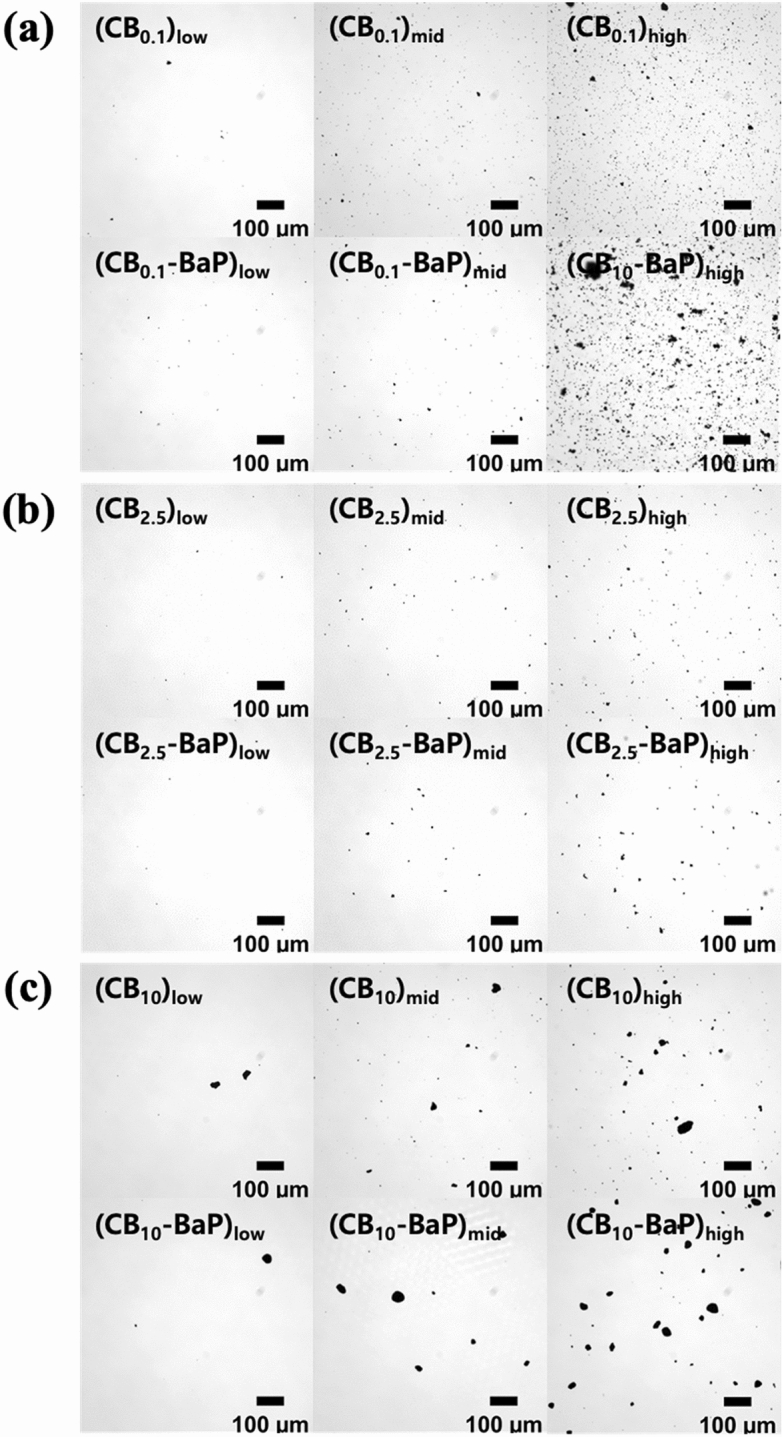
The microscopic images of particulate matters (PMs) generated through the spray drying process. The PMs were diluted with DMSO to make the low, mid, and high concentrations of PMs. (**a**–**c**) Three sizes of CB were used to produce PMs with/without BaP and three concentrations of PMs were prepared for the cell test. The titles in the images indicate the group names shown in Table 1. In all PM groups with BaP, particles are agglomerated relatively compared to single CB groups. Specially, in PM_10_ without BaP, small-sized particles and clustered particles are observed together as shown in (**c**).

**Figure 3:**
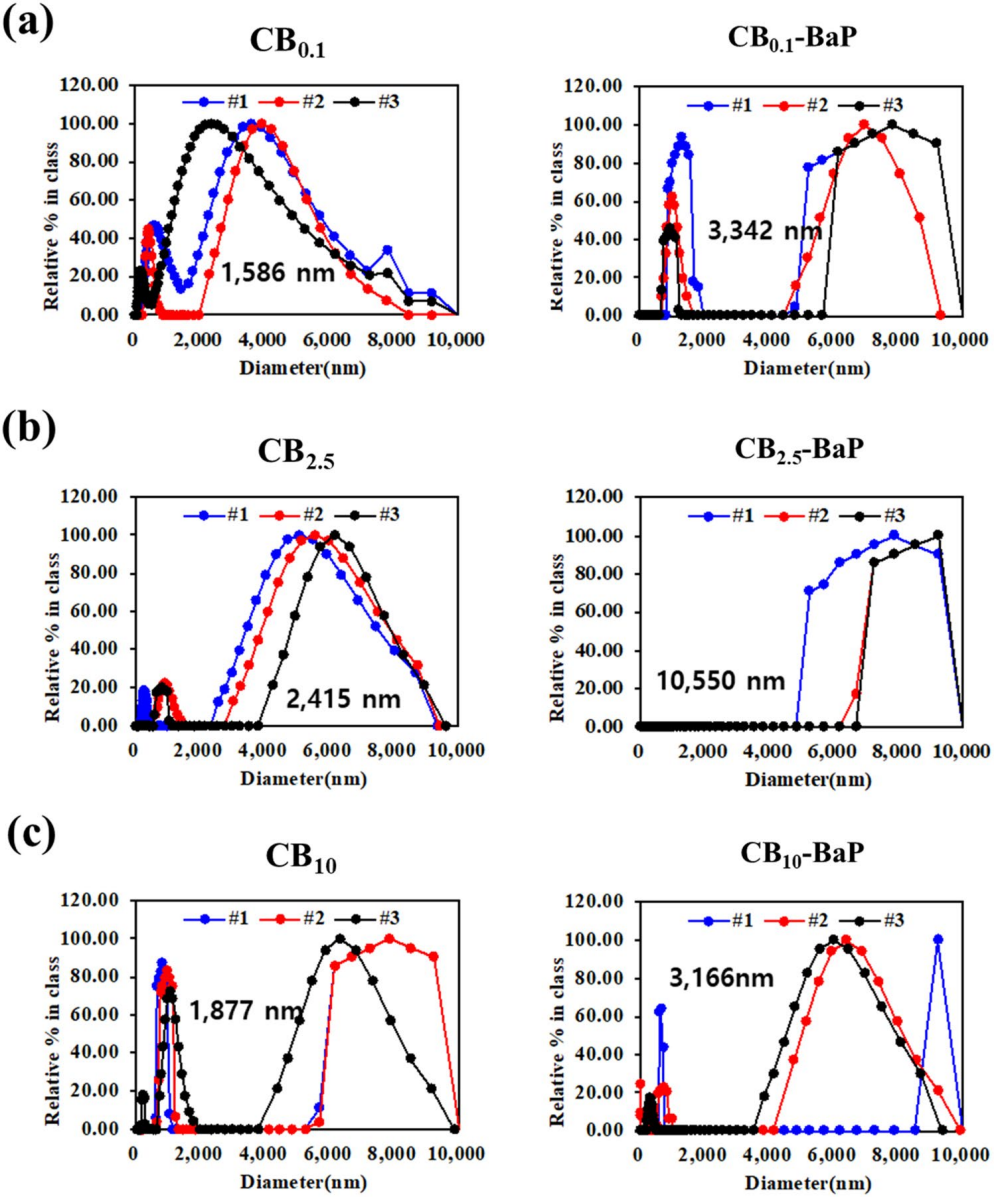
Dynamic light scattering (DLS) analysis for size distributions of PMs. Pannels (**a**)–(**c**) show the size distribution of PMs. According to the DLS measurement principle, if the sample concentration is too high, multiple scattering occurs and the signal-to-noise ratio increases. Thus, for accurate measurement of particle size distribution in suspension state, all group samples were prepared at the final concentration of 100 μg/ml with distilled water after dispersing the spray-dried particles in 100 μl DMSO. Finally, 3 ml samples were placed in a cuvette, and the DLS measurement was repeated three times.

**Figure 4:**
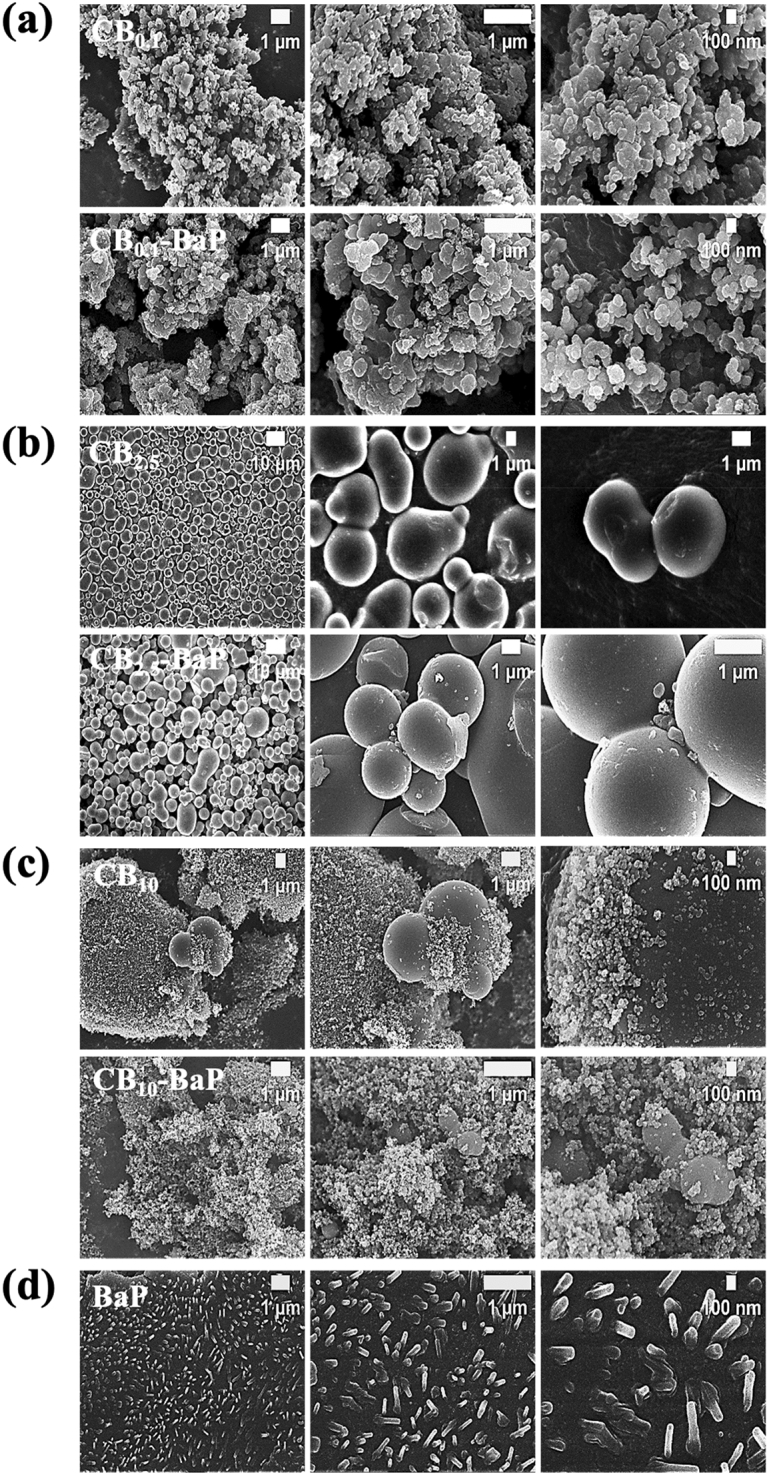
Scanning electron microscopy (SEM) images of PMs. The difference in the surface morphology of the particles according to the composition of PMs was shown in (**a**–**d**). In PM_0.1_, (**a**) indicates that small-sized particles are similarly aggregated together. In PM_2.5_, the presence of CB and BaP together, the particles clustered to form a relatively large agglomerate, as shown in (**b**). In PM_10_, (**c**) shows the agglomeration of particles of various sizes. Particularly, a lot of particles with a size of 100 nm were distributed like PM_0.1_.

**Figure 5:**
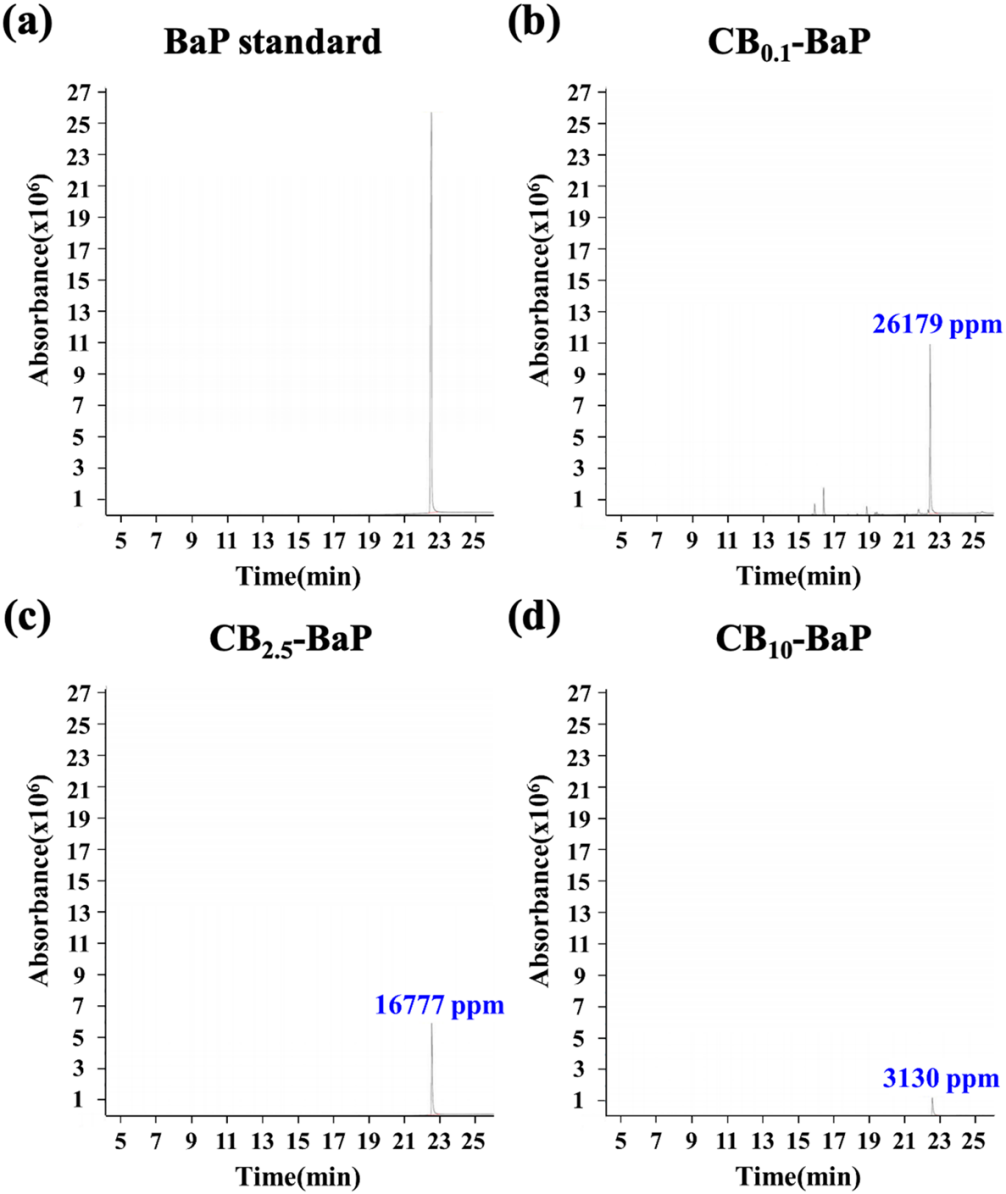
Gas chromatography–Mass Spectrometry (GC/MS) chromatograms of the BaP. The adsorption amount of BaP according to the size of CB was quantified. The *x*-axis of the graph represents the time benzopyrene passed through the column and reached the mass spectrometer detector, and the y-axis reflects the signal of benzopyrene abundance. (**a**) BaP standard, (**b**) CB_0.1_–BaP, (**c**) CB_2.5_–BaP, (**d**) CB_10_–BaP.

1. The morphology of the particles was confirmed using a microscope at the same concentration (10, 100, and 500 μg/ml) as PM exposed to A549 cells (Fig. 2).
2. DLS analysis was performed to determine the size distribution of the particles in suspension (Fig. 3).
3. The surface morphology of the CB and CB-BaP particulates was characterized using SEM (Fig. 4).
4. Gas Chromatography–Mass Spectrometry (GC–MS) analysis was performed to quantify the amount of adsorbed BaP according to the CB size (Fig. 5).

The aggregation of CB particles containing BaP at high concentrations of PM_0.1_, PM_2.5_ and PM_10_ was visually confirmed using microscopic images (Fig. 2). In PM_0.1_, PM_2.5_, and PM_10_, the average size of CB increased with BaP compared with that of CB alone (Fig. 3). Interestingly, CB_10_ contained many smaller-sized particles, resulting in two peaks in the DLS analysis (Fig. 3c). The electron micrographs in Fig. 4 show the size, shape, aggregation, and surface structure of PMs. PM_0.1_ showed little difference in surface morphology depending on the presence of BaP. However, the SEM images of CB_10_ confirmed that small particles, similar to PM_0.1_ were distributed on the CB_10_ surfaces, as shown in Fig. 4c.

In summary, the analysis of the physical properties of PMs showed that BaP promotes the aggregation of CB, increasing the PM size. In PM_0.1_, PM_2.5_, and PM_10_, the presence of BaP resulted in relatively larger particle sizes than those without BaP. In particular, we confirmed that the CB_10_ group had two different size ranges for PM_10_ and PM_0.1_, regardless of the presence of BaP. Because the spray-drying process provides a very harsh environment with high turbulence at high temperatures, collisions between particles and walls with viscous shear stress can break CB_10_ into smaller particles, similar to CBM_0.1_. It was confirmed that the small particles, which were formed as a result of the collisions and viscous shear stress, had a significant toxic effect, as evidenced by the low cell viability in the MTT assay results.

#### Chemical characteristics

As shown in Fig. 5, GC/MS analysis was performed to accurately determine the BaP content of the particles produced by controlling their size and components through spray drying. The BaP standard used was the same as that used in the experiments. The amount of time required for BaP to pass through the column and reach the mass spectrometer detector was approximately 22–23 min. In the CB_0.1_–BaP group, a high level of BaP was detected at 26,179 ppm (Fig. 5b). The BaP content was the highest in the order of CB_0.1_–BaP, CB_2.5_–BaP, and CB_10_–BaP; therefore, we confirmed that the amount of BaP loaded into the CB increased as the CB size decreased.

### Biological analysis

#### A549 cells exposed to PM

**Figure 6:**
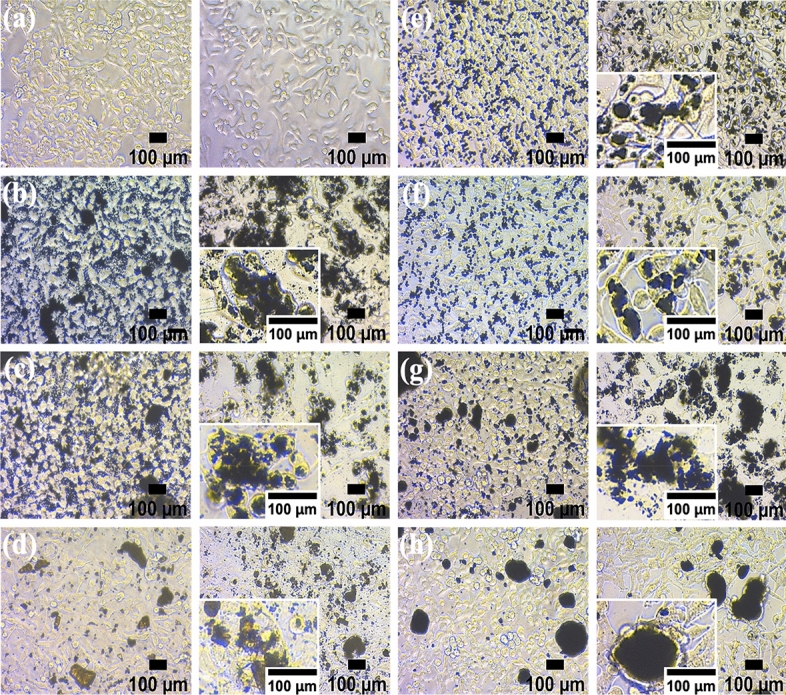
Microscopy images of human lung cells (A549) exposed to particulate matter (PM). The cells immediately after being exposed to 500 μg/ml of PMs (the left images). Morphological alterations regarding particles penetration and aggregation in A549 cells after 24-h exposure to the high concentration of PMs (the right images). (**a**) Control, (**b**) (CB_0.1_)_high_, (**c**) (CB_0.1_–BaP)_high_, (**d**) (BaP)_high_, (**e**) (CB_2.5_)_high_, (**f**) (CB_2.5_–BaP)_high_, (**g**) (CB_10_)_high_, and (**h**) (CB_10_−BaP)_high_. The black spots in the images indicate PMs applied to the cells.

To investigate the effects of PM on human A549 cells based on the size, concentration, and presence of BaP, we first examined cell viability using the MTT assay. After A549 cells were attached to the flask and stabilized, they were exposed to different concentrations of PM for 24 h (Table. 1). As shown in the left-side images of Fig. 6, we confirmed that the PM were uniformly suspended on A549 cells immediately after PM exposure. After 24-h exposure, in CB_0.1_, CB_0.1_-BaP, and CB_10_, the cell morphologies were degraded because the particles penetrated the cells and aggregated (Fig. 6b, c, g). In CB_2.5_, the particles infiltrated A549 cells, regardless of the presence of BaP (Fig. 6e, f). In CB_10_-BaP, the particle size was larger than that of the other CBs, and no infiltration of the particles into the cells was observed. Therefore, there was no significant difference in morphology from the control group (Fig. 6h).

#### MTT assay for cell viability

**Figure 7:**
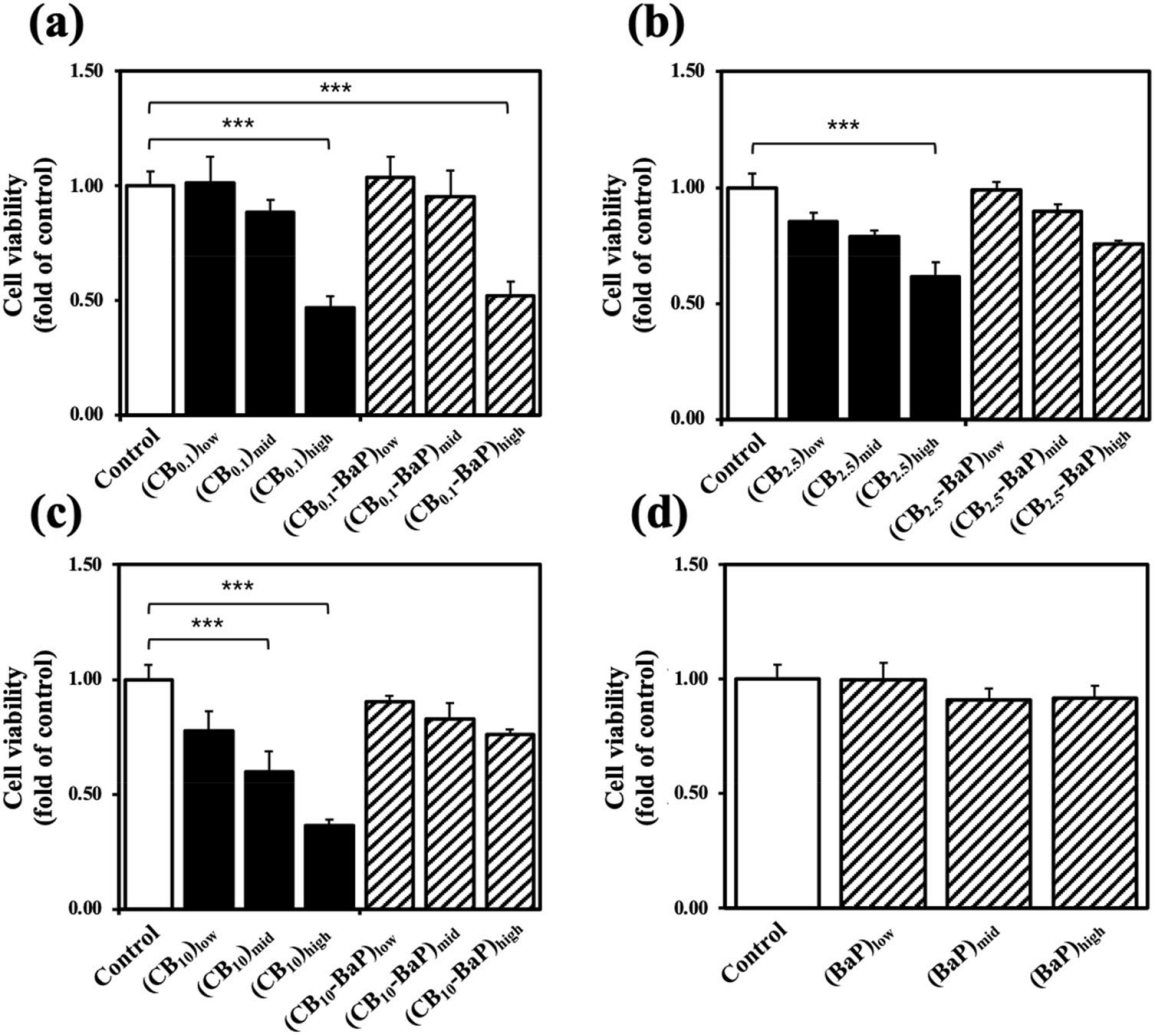
Viabilities of A549 cells exposed to particulate matters (PMs). (**a**) PM_0.1_, (**b**) PM_2.5_, (**c**) PM_10_, and (**d**) BaP. A549 cells were exposed to PMs with or without BaP for 24 h at PM concentrations of 10, 100, and 500 μg/ml. The quantification of the purple crystals formed after the MTT assay procedure reflects the viability of the cells. The absorbance of the colored solution is measured at 570 nm to determine the degree of cell survival. Cell viability was calculated as a ratio of the average viability of the control group to that of an experimental group. Values are the means ± standard error of the mean (n = 3). The statistical significance shown with the symbol of ***indicates that *p* < 0.001 compared with the control group.

The cytotoxicity assays (Fig. 7) showed the viability of A549 cells exposed to PM_0.1_, PM_2.5_, and PM_10_ composed of CB with/without BaP. Overall, a PM-concentration-dependent decrease in cell viability was observed. At the highest PM concentration (500 μg/ml), the lowest cell viability was observed in all PM groups, and CB_0.1_ and CB_0.1_-BaP showed similarly low survival rates of 47% and 52%, respectively, regardless of BaP. Interestingly, the lowest survival rate of 37% was obtained in CB_10_ without BaP owing to the dual range of particle sizes shown in Figs. 3c and 4c. In addition, the cell viabilities in the PM_0.1_, PM_2.5_, and PM_10_ groups were lower when exposed to CB alone than when exposed to CB-BaP. This result indicated that the increase in PM size, attributed to the presence of BaP, mitigated the decrease in cell viability. The group exposed to BaP alone did not show significant apoptosis during the day. In other words, there was no immediate inflammatory response in A549 cells to BaP. As a result, significant cytotoxicity was observed in CB_0.1_ and CB_10_ at 500 μg/ml.

#### Generation of ROS

**Figure 8:**
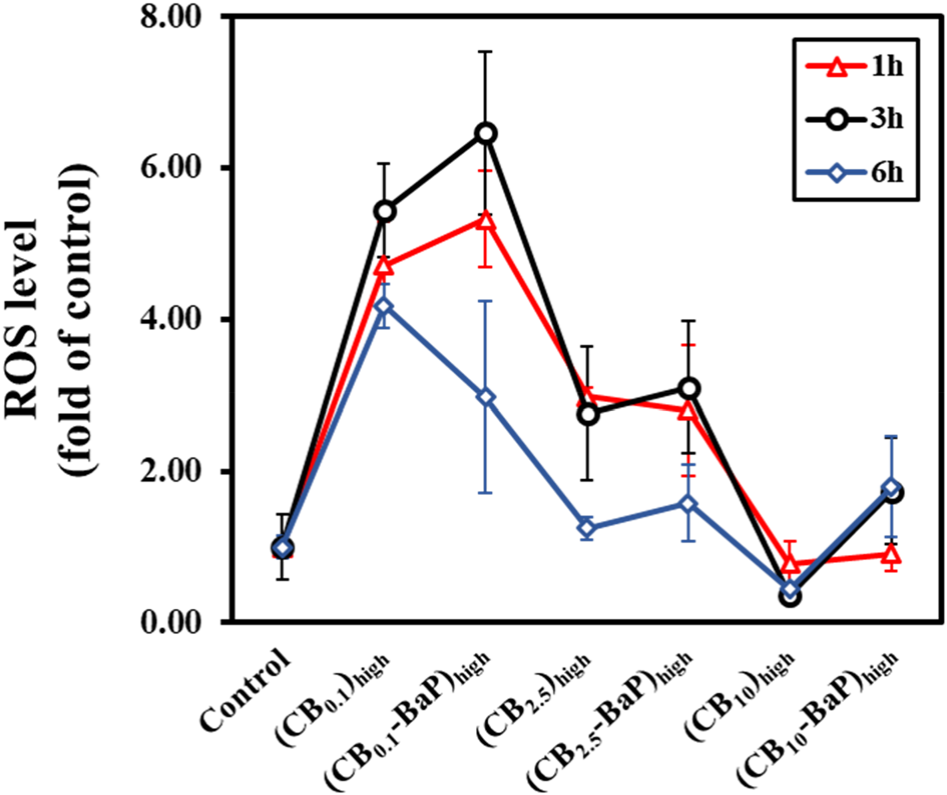
Assessment of reactive oxygen species (ROS) production in live A549 cells exposed to PMs. The cells were incubated for one, three, and six hours after exposure. The *x*-axis represents the high concentration of PM experimental groups shown in Table 1, and the *y*-axis indicates the relative DCF fluorescence intensity formed by ROS to the control. Fluorescence quantification was measured using a microplate reader with excitation and emission wavelengths at 485 and 535 nm.

High oxidative stress induces DNA damage that is associated with cell death. In addition, oxidative stress causes damage to mitochondrial DNA due to 8-Oxoguanosine accumulation and failure to repair DNA. Furthermore, the occurrence of ROS, such as cell pre-destination, is directly related to cell survival^28^. The intracellular levels of ROS were measured by the fluorescence of DCFH-DA probe formation in A549 cells after exposure to PM_0.1_, PM_2.5_, and PM_10_ at a high concentration of 500 μg/ml for 1 h, 3 h, and 6 h. From the viewpoint of physical properties, an increase in ROS levels was obtained with a decrease in PM size, as shown in Fig. 8. In addition, the CB-BaP groups showed higher ROS levels than the CB group in the early stage of exposure between 1 and 3 h; however, the ROS levels of the CB-BaP groups rapidly decreased 6 h after exposure. For ROS measurements, the PM exposure time was up to 6 h according to the assay protocol, so it was not possible to observe ROS release according to the exposure time thereafter. Therefore, PM_10_ generates ROS after 6 h and tends to be slightly different from the results of MTT and ELISA assays, which are methods used to confirm the results after exposure to PM for 24 h. These findings suggest that PM_0.1_ is the most associated with cytotoxicity, as shown by the MTT assay results. We found that the size of PMs was a significant factor in determining the ROS level and accelerating the effect of BaP on the release of ROS.

#### Activation of the pro-inflammatory response (ELISA)

**Figure 9:**
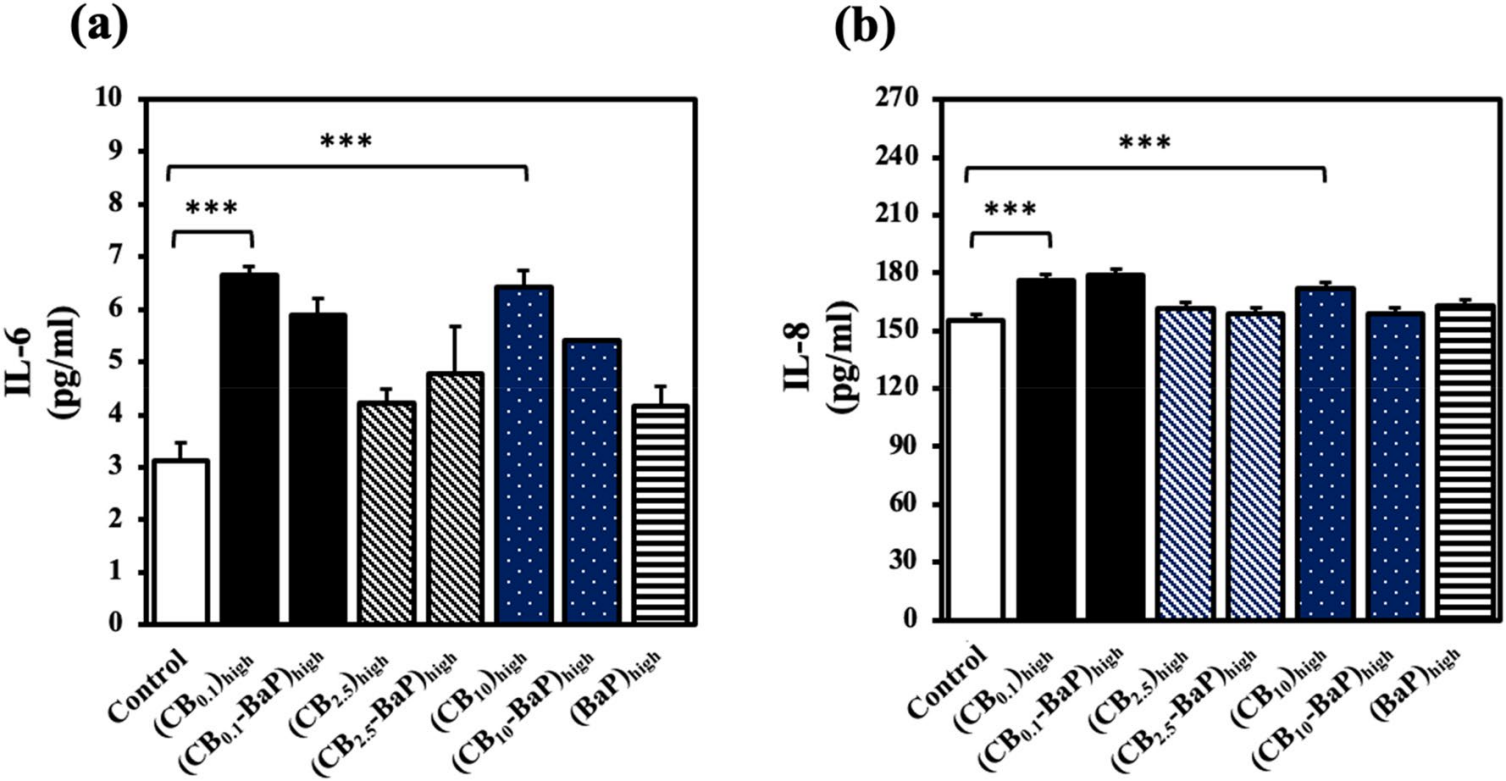
Expressions of pro-inflammatory cytokines from A549 cells exposed to PMs. Differentiated A549 cells were exposed to PMs for 24 h, and the Sandwich ELISA was used to evaluate inflammatory cytokine levels. The IL-6 and IL-8 levels induced from the cell supernates were measured by detecting colorimetric changes. To measure the relative amounts of IL-6 and IL-8 in A549 cells exposed to PMs, standard curve graphs were obtained using human recombinant IL-6 and IL-8 as standard substances in the kit, and cytokines were quantified. In the IL-8 assay procedure, the cell supernates were diluted 100-fold because the concentration of antigens exceeded the highest standard value. The error bars represent ± standard error of the mean (n = 3). The statistical significance differences marked with *** indicate *p* < 0.001 compared with the control group.

A sandwich-based ELISA method was used to examine the release of pro-inflammatory proteins upon PM exposure in the supernatants of A549 cells^29,30^. A549 cells were exposed to high concentrations of PM_0.1_, PM_2.5_, and PM_10_ (500 μg/ml) for 24 h. The IL-6 and IL-8 levels in the PM_0.1_ group were higher than in the other groups (Fig. 9). As shown in Fig. 8, the ROS levels of PM_0.1_ and PM_10_ significantly increased compared to those in the control group, but the presence of BaP did not considerably affect the ROS level. Consequently, we ascertained that particle size had a more significant effect on the secretion of inflammatory cytokines in human lung cells than BaP.

## Discussion

Our results demonstrated that the effect of particle size, rather than the presence or absence of BaP, was a major factor in the cytotoxic effect. In this study, CB_0.1_, CB_2.5_, and CB_10_ were coated with BaP to prepare PMs of controlled sizes and compositions. This was tested to determine how changes in the particle properties that occur when BaP is adsorbed onto CB affect A549 cells.

### Physicochemical characteristics of PMs

The toxicological activity of PMs differs according to its size, concentration, and chemical composition. Several studies have shown that particle toxicity is mainly determined by particle size^10^. In addition, among the representative components of PMs, studies on the harmfulness of CB and PAHs have been conducted separately^31^. However, the combined toxicity from physicochemical properties is not well understood. Accordingly, our research was conducted by independently controlling both PM size and chemical composition. Our study aimed to evaluate the effect of PMs as a function of their physicochemical properties on the inflammatory response in A549 cells.

Physicochemical analysis confirmed that BaP was adsorbed on the carbonaceous core, causing agglomeration, thereby increasing the size of the PMs as shown in Fig. 2^21^. We demonstrated the size distribution of PM0.1 and PM10 particles for each group using DLS, as shown in Fig. 3a, c. We confirmed that the CB_10_ group had two points of size distribution, indicating that it contained relatively more particles of smaller sizes compared to PM_0.1_. This observation suggests that this smaller part Regarding CB10, the study showed that in the absence of BaP, the higher toxicity observed could be attributed to the presence of smaller particles compared to CB_0.1_. However, in the presence of BaP, the toxic effect was found to be lower for PM_10_ than PM_0.1_, possibly due to the agglomeration effect of BaP, which reduced the smaller particle effect. Therefore, it can be concluded that the absence or presence of BaP affects the toxic effect of CB_10_ differently depending on the size distribution of the particles. In addition, it was identified that the smaller the CB size, the more BaP was adsorbed because of the increase in the surface area of the particles (Fig. 5). This means that the smaller the PMs, the greater the amount of BaP delivered to the cells^20,21^. As shown in the results of previous studies^17,18^, we confirmed that a large amount of PAH was adsorbed onto smaller-sized carbon in A549 cells, leading to a greater delivery of toxicity. As the concentration of BaP increases, toxicity also increases, and thus the toxic effect of BaP can be higher in small particles. our study discovered a new effect of BaP, which is the mechanism of increasing the PM size. By explaining the effect of BaP in particles of the same size through this newly discovered mechanism, we have provided an explanation for BaP's effect^17^. Finally, as a result of fabricating and analyzing particles by independently controlling the size of PMs and the chemical composition of BaP using CB, we discovered that the chemical properties of the particles greatly contributed to determining the final size of PMs. Therefore, it was confirmed that the change in particle size caused by adsorption onto CB was greater than that of BaP.

From our results, it was identified that the smaller the size of CB, the more BaP was adsorbed because of the increase in the surface area of the particles. In the presence of BaP in CB, it was observed that the particles agglomerated and increased in size in PM_0.1_, PM_2.5_, and PM_10_. One unanticipated finding was the distribution of particles of the same size as PM_0.1_ CB_10_-BaP, as shown in Figs. 3c and 4c. Accordingly, it was perceived that BaP adsorbed on CB induces physicochemical changes in the particles, and an increase in particle size reduces biological effects.

### Biological effects of unloaded and BaP-loaded CB

As shown in Fig. 7, the cell viabilities measured using the MTT assay showed a tendency to decrease depending on the concentration of the particles exposed to A549 cells. Because BaP increased the size of PM, the cell viability of the CB-BaP group was higher than that of the single CB. Even though the cell survival rate was most conspicuously reduced in CB_10_, the cell survival rate was also low in PM_0.1_ regardless of the presence or absence of BaP. The reason for the lower cell viability in PM_10_ than in PM_0.1_ is that the physicochemical analysis results of PMs (Figs. 3c and 4c) exhibit a distribution of small-sized particles such as PM_0.1_. In addition, A549 cells exposed to PM_0.1_ and CB_10_ showed similar cell morphology and degree of degradation after 24 h, as shown in Fig. 6b, c, g. Although PM_2.5_ did not significantly affect the cytotoxic effect, A549 cells exposed to PM_2.5_ showed that the particles were absorbed into the cells through endocytosis^32^.

The production of intracellular ROS induced by PMs was evaluated by measuring the actual levels of ROS with H2CFDA. ROS production was evident at the initial exposure time (1 h, 3 h, and 6 h). The highest ROS level of PM_0.1_, suggests that ROS generation also affects cytotoxicity, but is more significantly affected by particle size in Fig. 8. Mitochondria are the main sites of ROS generation, and the smaller the PM, the easier it is to access the mitochondria, causing damage and inducing apoptosis^33^. This suggests that PM_0.1_-induced ROS production can contribute to cell apoptosis by activating NF-κB and AKT/STAT3 phosphorylation in lung epithelial cells^34–36^. This effect was also mediated by upregulation of IL-6 expression. The reason for the low ROS level in CB_10_, unlike the cell viability measurement result, is that the exposure time to A549 cells and the measurement time are different^37^. The cell viability results were measured after 24 h of PM exposure, while ROS was evaluated up to a maximum of 6 h because of the time limit of the company's result guarantee. Therefore, the tendency of cell viability and ROS may differ due to the variance in evaluation time. ROS results can be considered to indicate the cause of PM toxicity for initial exposure within 6 h after PM exposure. According to the ROS results, it can be clearly confirmed that the smaller the size of the initial PM exposure, the higher the ROS level. Interestingly, PM10 shows a tendency for a slower decrease in ROS levels, and PM_10_-BaP shows an increase in ROS levels over time. In conclusion, the size of PM can be considered a significant factor influencing the ROS level during the initial exposure to PM. In association with the cell viability results, it can be inferred that smaller PM sizes rapidly increase ROS levels from the initial exposure, leading to a severe decrease in cell viability.

Additionally, the results of the ROS experiment showed that A549 cells exposed to PM_0.1_ and CB_10_ did not exhibit similar results, unlike in other experiments. Therefore, these ROS experiment results appear to have a different mechanism of action compared to the results from other biological analyses.

PMs directly stimulate macrophages and lung epithelial cells to produce inflammatory cytokines such as IL-6 and IL-8. IL-6 is promptly and transiently produced in response to cell DNA damage and is associated with the exocytosis of macrophages and mast cells^38^. IL-8 is the most potent activator and chemoattractant for neutrophils, eosinophils, and T lymphocytes, causing lung inflammatory reactions^39^. In our study, higher levels of inflammatory cytokines were secreted in all experimental groups than in the control group. However, the presence of BaP in the PM_2.5_ group was found to cause a slight change in the level of IL-6. Additionally, high levels of cytokine expression were observed in PM_10_ without BaP, similar to PM_0.1_. These results are consistent with the findings of cytotoxicity and oxidative stress analyses. This study found that the toxicity of PM varies depending on the time frame and whether it is caused by chemical or physical effects. Specifically, CB_0.1_ was found to have higher levels of ROS over time compared to CB_0.1_-BaP. However, it was not possible to measure ROS levels over a longer period of time due to methodological limitations. There are also experimental limitations in using cell viability results after 24 h to confirm this difference. The time-dependent toxicity effect goes beyond the scope of this paper, and further research is needed to investigate it. Increases in IL-6 and IL-8 protein expression were similarly observed in PM_0.1_ regardless of the presence of BaP. These results are similar to those of the cytotoxicity and oxidative stress analyses. Therefore, our studies revealed that the physical properties (physical toxicity) of PMs can be a dominant factor in determining the release of PM-induced cytokines rather than the biological hazards (chemical toxicity) of substances adsorbed to PMs.

**Figure 10:**
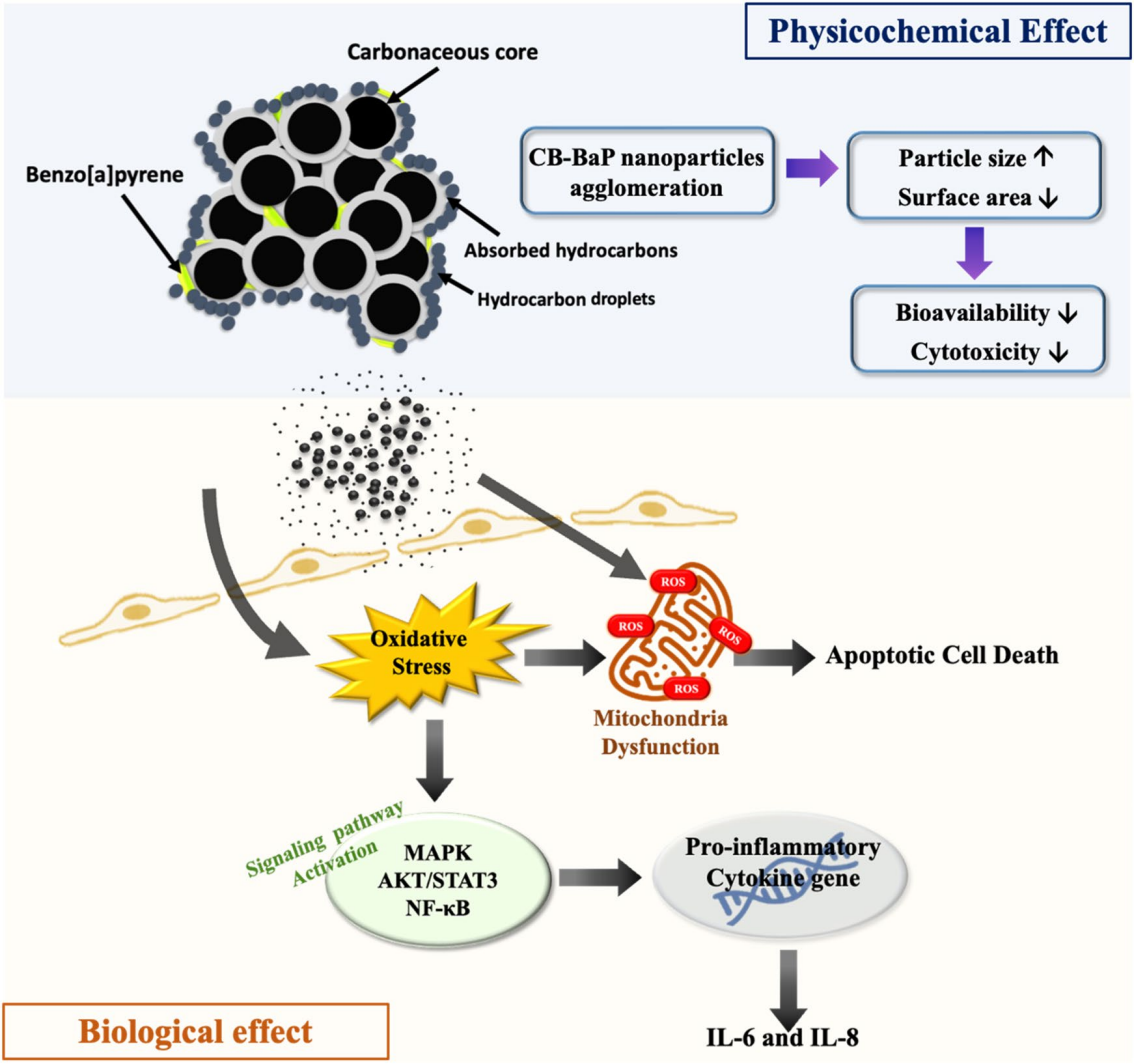
The mechanisms of inflammatory response in human lung cells caused by the physicochemical properties of PM composed of carbon black (CB) and benzo[a]pyrene (BaP). When BaP is adsorbed on the CB particles, the effective particle size increases due to the aggregation enhancement by BaP. As a result, the overall surface area of CB particles decreases, reducing cell biological availability and cytotoxicity. The influx of particles into the cells primarily damages cell membranes, induces oxidative stress, and activates signal transduction pathways triggering apoptosis. First, IL-6 and IL-8, which are inflammatory cytokines, are released, and second, ROS is generated by inducing oxidative stress in mitochondria. When mitochondrial dysfunction occurs, DNA, protein, and lipids are permanently damaged, leading to cell death.

Based on a comprehensive analysis of the physicochemical properties of PMs and the biological responses of human lung cells, we discovered the biological effects a new role and mechanism of BaP adsorbed on CB particles in the inflammatory response of the lung cells, as shown in Fig. 10.

## Conclusion

This study examined the physicochemical properties of PM consisting of CB and BaP in human lung epithelial cells. The size, chemical composition, and concentration of PMs are potential contributors to cytotoxicity, and the combination of these factors complicates their biological effects. To ascertain the instant interaction of PMs with specific components on cytotoxicity-related impacts, the size and chemical composition of PMs were independently controlled through the aerosolization process, and human lung cells were exposed to the PMs at different concentrations for one day.

Our results showed that the size of PMs contributed more significantly to the cytotoxicity effect than the presence of BaP adsorbed on the CB surfaces. However, the ROS assay did indicate an increase in ROS upon BaP addition, suggesting that BaP may have other mechanisms that contribute to cytotoxicity^40^. We found that BaP played a vital role in increasing the PM size, and the amount of BaP loaded into the CBs increased with decreasing PM size. Interestingly, PMs containing BaP alone did not cause significant cell damage in a single day. Additionally, Zarei et al. demonstrated the cytotoxic effects of BaP on A549 cells by subjecting the cells to a 72-h exposure and assessing the cellular viability using an MTT assay^41^. Therefore, we will investigate the long-term cytotoxic effects and mechanisms of exposure to PMs in future studies. The study observed that PM_0.1_ showed the most significant inflammation response regardless of the presence of BaP. PM_0.1_ caused an immunotoxic effect by increasing the oxidative stress and pro-inflammatory mediated responses of the cells due to the increase in contact area with the PMs. Similarly, the study observed that even in the CB_10_ group with a size distribution similar to PM0.1, small particles had cytotoxic effects on A549 cells. In contrast, PM_2.5_ and CB_10_-BaP showed relieved inflammatory responses, especially when they were formed with BaP due to the size increase. The ROS assay revealed that A549 cells exposed to PM_0.1_ and CB_10_ did not exhibit similar results compared to the other biological analyses. Thus, the ROS assay findings suggest a different mechanism compared to the other assays. This study provides a new understanding of the role of BaP in varying the physicochemical characteristics of PMs and the toxicological effects on human cells. Thus, the ROS assay findings suggest a different mechanism compared to the other assays. This study provides a new understanding of the role of BaP in varying the physicochemical characteristics of PMs and the toxicological effects on human cells.

We will further investigate the long-term effects of PMs with different physical and chemical properties on the inflammatory response in mouse lungs (in vivo) to overcome the limitation of exposure time for in vitro experiments. The research provides fundamental indicators for future work on the biological impacts of different physicochemical properties of PMs and will lead to new strategies for reducing the most critical factors in air pollution by identifying the toxic transmission routes of PMs. The size of PMs determines cell degradation through the penetration of cells by endocytosis. In addition, the amount of BaP loaded into the CBs increased with decreasing PM size. However, interestingly, PMs containing BaP alone did not cause significant cell damage in a single day. As a result, PM_0.1_ showed the most significant inflammation response regardless of the presence of BaP. PM_0.1_ caused an immunotoxic effect by increasing the oxidative stress and pro-inflammatory mediated responses of the cells due to the increase in contact area with the PMs. Similarly, we observed that even in the CB_10_ group with a size distribution similar to PM_0.1_, small particles had cytotoxic effects on A549 cells. In contrast, PM_2.5_ and CB_10_-BaP showed relieved inflammatory responses, especially when they were formed with BaP due to the size increase. This study found that the toxicity of PM varies depending on the time frame and whether it is caused by chemical or physical effects. Specifically, CB_0.1_ was found to have higher levels of ROS over time compared to CB_0.1_-BaP. However, it was not possible to measure ROS levels over a longer period of time due to methodological limitations. There are also experimental limitations in using cell viability results after 24 h to confirm this difference. The time-dependent toxicity effect goes beyond the scope of this paper, and further research is needed to investigate it. In contrast, PM_2.5_ and PM_10_ showed relieved inflammatory responses, especially when they were formed with BaP due to the size increase. Accordingly, our study provides a new understanding of the role of BaP in varying the physicochemical characteristics of PMs and the toxicological effects on human cells.

We will further investigate the long-term effects of PMs with different physical and chemical properties on the inflammatory response in mouse lungs (in vivo) to overcome the limitation of exposure time for in vitro experiments. This research provides fundamental indicators for future work on the biological impacts of different physicochemical properties of PMs. Furthermore, our findings will lead to new strategies for reducing the most critical factors in air pollution by identifying the toxic transmission routes of PMs.

## Data Availability

The data supporting the findings of this study are available upon request from the corresponding author.

## Abbreviations

PM: Particulate matter
CB: Carbon black
BaP: Benzo[a]pyrene
PAHs: Polycyclic aromatic hydrocarbons
DLS: Dynamic light scattering
DMSO: Dimethyl sulfoxide
CB: Carbon black
GC–MS: Gas chromatograph–mass spectrometer
ROS: Reactive oxygen species
